# Unique Molecular Characteristics of Pediatric Myxopapillary Ependymoma

**DOI:** 10.1111/j.1750-3639.2009.00333.x

**Published:** 2010-05

**Authors:** Valerie N Barton, Andrew M Donson, Bette K Kleinschmidt-DeMasters, Diane K Birks, Michael H Handler, Nicholas K Foreman

**Affiliations:** 1 Departments of Pediatrics, Anschutz Medical Campus, University of Colorado DenverAurora, Col.; 2 Departments of Neurosurgery, Anschutz Medical Campus, University of Colorado DenverAurora, Col.; 3 Departments of Pathology and Neurology, Anschutz Medical Campus, University of Colorado DenverAurora, Col.; 4 The Children's HospitalDenver, Aurora, Col.

**Keywords:** HOX, microarray, myxopapillary ependymoma, NEFL, PDGFRα, pediatric neuro-oncology

## Abstract

Myxopapillary ependymoma (MEPN) generally can be cured by gross total surgical resection and usually manifest a favorable prognosis. However, surgery is less curative in tumors that are large, multifocal or extend outside the thecal sac. Late recurrences may occur, particularly in pediatric patients. The role of adjuvant therapy is unclear in the clinical management of recurrent tumors. Clinical trial design requires a better understanding of tumor biology. Unique molecular features of MEPN were investigated by using microarray technology to compare the gene expression of five pediatric MEPN to 24 pediatric intracranial ependymoma (EPN). The upregulation of three genes of interest, homeobox B13 (HOXB13), neurofilament, light polypeptide (NEFL) and PDGFRα, was further studied by immunohistochemistry in a larger cohort that included adult MEPN and EPN specimens. Protein expression in MEPN was compared to subependymoma, spinal EPN, intracranial EPN and normal fetal and adult ependyma. Immunoreactivity for HOXB13, NEFL and PDGFRα was strongest in MEPN and virtually absent in subependymoma. Spinal and intracranial EPN generally expressed weak or focal staining. MEPN manifests unique gene and protein expression patterns compared to other EPNs. Aberrant expression of HOXB13 suggests possible recapitulation of developmental pathways in MEPN tumorigenesis. PDGFRα may be a potential therapeutic target in recurrent MEPN.

## INTRODUCTION

Myxopapillary ependymoma (MEPN) is a slow-growing ependymoma type that is virtually confined to the conus medullaris-cauda equina-filum terminale region of the spinal cord (8). Histologically, MEPN is characterized by a papillary arrangement of tumor cells around vascularized myxoid stromal cores and corresponds to World Health Organization (WHO) grade I. MEPN constitutes approximately 13% of ependymoma (EPN) (32). Age of diagnosis ranges from 6 to 82 years with an average age of 36.4 years (10). Standard treatment of MEPN is aggressive surgery. However, tumor recurrence can occur with incomplete resection and the role of adjuvant chemotherapy (25, 36, 37) or radiotherapy (30, 33, 41) is unresolved for the subset of patients with recurrence or in patients in whom gross total resection cannot be achieved.

Pediatric patients are more likely to develop metastases and display a more aggressive course of disease (11, 16, 17, 22). A recent study by Bagley *et al*. examined the clinical course of 52 MEPN including 14 pediatric patients and 38 adult patients (5). Pediatric patients had a higher rate of local recurrence and tumor dissemination within the neural axis (64% compared to 32%). Although these numbers are higher than previously reported in the literature, they are likely to be more accurate due to the study's long-term follow-up (11.5 years). No benefit for adjuvant chemotherapy or radiation therapy was demonstrated.

Clinical trial design for novel adjuvant therapies mandates better knowledge of the biology of MEPN. Few studies have examined the molecular biology of MEPN compared to intracranial and spinal EPN tumors. In a study of 62 ependymal tumors including six MEPN, Ebert *et al* found six NF2 mutations in grade II spinal EPN but no mutations in MEPN (14). Santi *et al* investigated chromosome 7 copy number in 27 adult EPN, including 13 MEPN, by chromogenic *in situ* hybridization. All 13 of the MEPN tested displayed polysomy of chromosome 7 in contrast to the other EPN, which were diploid (31). A study by Lukashova-v Zangen *et al* compared the gene expression of eight MEPN and six subependymomas (SEPN) by cDNA microarray and real-time polymerase chain reaction (21). They reported 30 genes that were more highly expressed in SEPN than MEPN including ETV6, YWHAE, TOP2A, TLR2, ADE2H1, IRAK1, TIA1, TTL, UFD1L, TOMM70A and HSD3B1. Unique molecular characteristics of MEPN were not described. Finally, Korshunov *et al* examined 39 ependymal neoplasms including four MEPN by microarray (19). MEPN were found to be molecularly distinct from intracranial EPN, with high expression of HOXB5, PLA2G5 and ITIH2. However, the study reported only three genes of interest and did not confirm microarray results with protein expression data.

The aforementioned studies suggest that MEPN is molecularly distinct from other types of EPN but provide few clues into the biology of these tumors. Novel therapies may be especially pertinent to pediatric MEPN, which have a more aggressive course of disease that is clinically difficult to control despite the deceptively “benign” WHO grade I designation. The present study uses microarray and immunohistochemistry (IHC) to compare the biology of pediatric MEPN and intracranial EPN to identify unique molecular characteristics of MEPN and potential therapeutic targets.

## METHODS

### Study cohort

A retrospective analysis was performed on tumor specimens obtained from The Children's Hospital, Denver or University of Colorado Hospital. All studies were conducted in compliance with local and federal human research protection guidelines and institutional review board regulations (COMIRB #95-500 and #08-0944).

Gene expression microarray analysis was conducted on five pediatric MEPN and 23 pediatric intracranial EPN. In addition to the ependymoma variants, 50 other pediatric central nervous system (CNS) tumor specimens were analyzed by microarray including 18 glioblastomas (GBM), 10 pilocytic astrocytomas (PA), nine atypical teratoid/rhabdoid tumors (ATRT), nine classical medulloblastomas (MED) and four large-cell medulloblastomas (LCM).

IHC specimens included 13 MEPN (five pediatric, eight adult), eight spinal EPN (one pediatric, seven adult), 12 intracranial EPN (seven supratentorial, five infratentorial; seven pediatric, five adult) and five adult SEPN. The diagnosis, age, gender and grade of tumors included in the IHC study are summarized in supporting information Table S1. Conus medullaris and filum terminale spinal cord sections from five adult patients who did not suffer from neurological disease were included in the analysis. Spinal cord sections from three fetuses of 18, 23 and 35 weeks' gestation were also included in the analysis.

### Gene expression microarray analysis

Patient tumor samples were evaluated for gene expression using Affymetrix U133 Plus2 GeneChip microarrays (Santa Clara, CA, USA). Samples were collected at the time of surgery and snap-frozen in liquid nitrogen. RNA was extracted from each sample using an RNeasy kit (Qiagen, Valencia, CA, USA) according to the manufacturer's instructions and RNA quality was measured using a 2100 BioAnalyser (Agilent, Santa Clara, California USA). RNA was processed as described previously (12) and hybridized to HG-U133 Plus2 GeneChips (Affymetrix) according to the manufacturer's instructions. Microarray data from the samples was background-corrected and normalized using the gcRMA algorithm (45). One probe set per gene, based on highest overall expression level across samples, was selected for use in subsequent analyses. Differential expression of genes was determined using a Student's *t*-test. The false discovery rate, as defined by Benjamini *et al*., was set at <0.05 for all *t*-tests (7).

For clustering, data were filtered to include only genes that showed at least moderate expression (expression value greater than 5) and a range of expression values across all samples (only the top one-third of genes as ranked by the expression range) were used for the clustering analysis. These criteria resulted in the selection of 6414 genes. The hierarchical clustering used an agglomerative algorithm with average linkage and Euclidean distances, as implemented in the hclust package (35). Relative statistical strength of the resulting dendrogram branches was estimated using multiscale bootstrap resampling to estimate *P* values for each branch, based on 1000 replications, as implemented in pvclust. All R packages used are available through Bioconductor (http://www.bioconductor.org) or CRAN (http://cran.r-project.org).

### Immunohistochemistry (IHC)

IHC was performed on paraffin-embedded tumor tissue sections. All sections were immunostained in batch fashion. Slides were deparaffinized and antigen retrieval was performed using LPH Buffer (BioCare Medical, Concord, CA, USA) for 60 minutes at 93°C followed by a 20-minute cool down. Subsequent steps were performed using the EnVision-HRP kit (Dako, Glostrup, Denmark) on a Dako autostainer according to the standard protocol. Incubation with primary antibody was performed for 2 h. Antibodies for IHC included rabbit polyclonal anti-PDGFRα at a 1:200 dilution (sc-338; Santa Cruz Biotechnology, Santa Cruz, CA, USA), rabbit monoclonal anti-neurofilament, light polypeptide (NEFL) at a 1:100 dilution (2837S; Cell Signaling Technology, Danvers, MA, USA), and mouse monoclonal anti-homeobox B13 (HOXB13) at a 1:200 dilution (sc-28333; Santa Cruz Biotechnology).

Immunostaining was scored by the neuropathologist (BKD), who was blinded to the diagnosis or specific case number. All scoring was conducted twice over a several-day interval for concordance. After reviewing the slides in blinded fashion, the code was broken and slides were re-grouped according to histological diagnosis for the final review. The overall impression was the same as the original review, but this allowed direct comparisons between cases with similar diagnoses. Immunostaining was scored from − to ++ on a subjective scale. Twenty-five percent to 100% of cells staining yielded a score of ++. Very focal staining in individual cells yielded a score of +. Absence of staining was given a − designation.

### Statistical analysis

Statistical calculations were analyzed with R (http://cran.r-project.org/), Bioconductor (http://www.bioconductor.org) and Prism statistical analysis program (GraphPad Software, Inc., San Diego, CA). For all tests, a level of *P* < 0.05 was considered statistically significant.

## RESULTS

### Patient demographics

Patient details including diagnosis, age at diagnosis, gender and tumor grade are summarized in supplemental information Table S1. Briefly, the median age of pediatric MEPN patient diagnosis was 13 (ages ranged from 12 to 17 years). The male to female ratio of pediatric MEPN was 1:4. The median age at diagnosis was 3 years for pediatric intracranial EPN (age ranged from 0.5 to 18 years). Fifty-two percent of pediatric intracranial EPN corresponded to WHO grade II and 48% were grade III. Seventy percent of pediatric EPN were infratentorial and 30% were supratentorial. The male to female ratio was 1.3:1.0.

### Gene expression microarray in MEPN compared to intracranial EPN

**Figure 1:**
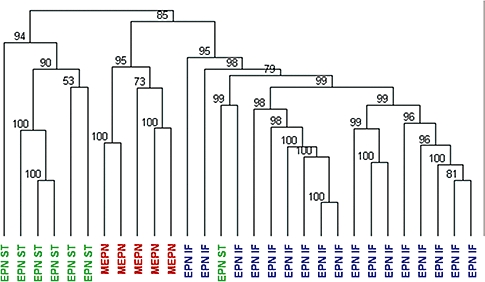
Hierarchical clustering analysis of the myxopapillary subgroup of ependymoma. Gene expression profiles of first presentation surgical samples of five pediatric MEPN and 24 pediatric intracranial EPN. Numerical values represent approximately unbiased *P* values that were computed by multiscale bootstrap resampling. Abbreviations: EPN = ependymoma; MEPN = myxopapillary ependymoma; IF = infratentorial; ST = supratentorial.

A clustering analysis of EPN gene expression profiles showed that pediatric MEPN (n = 5) formed a distinct subgroup from pediatric intracranial EPN (n = 24) (Figure 1). This analysis suggests that gene expression of MEPN is distinct from other intracranial EPN. Supratentorial and infratentorial EPN further divided into distinct subgroups. The association between molecular signature and tumor location in intracranial EPN has previously been reported by Modena *et al*(23).

Table 1 displays the top 15 over- and underexpressed genes in MEPN compared to intracranial EPN. Genes are ranked accorded to statistical significance. The most upregulated genes included small nuclear protein (PRAC); HOXB13; adp-ribosylation factor-like 15 (ARL15); homeobox C10 (HOXC10); and neurofilament light polypeptide 68 kDa (NEFL). Downregulated genes included amyloid beta precursor protein-binding, family a member 2 (APBA2); glycoprotein m6a (GPM6A); megalencephalic leukoencephalopathy with subcortical cysts 1 (MLC1); ankyrin repeat and btb domain containing 2 (ABTB2); and ring finger protein 43 (RNF43).

**Table 1:** Top 15 over- and underexpressed genes in pediatric myxopapillary versus pediatric intracranial ependymoma. Genes are ranked according to greatest statistical significance. In addition to the *P* values, *q* values are listed as a measure of the false discovery rate which estimates the probability of a false positive finding. The probe ID identifies the Affymetrix U133 Plus2 GeneChip probe set.

Gene symbol	Gene name	Probe ID	*P* value	*q* value
Genes overexpressed in myxopapillary ependymoma				
PRAC	Small nuclear protein	230784_at	1.00E-34	1.92E-30
HOXB13	Homeobox B13	209844_at	7.78E-33	7.49E-29
ARL15	Adp-ribsolyation factor-like 15	219842_at	1.47E-24	9.43E-21
HOXC10	Homeobox c10	218959_at	3.64E-24	1.40E-20
NEFL	Neurofilament, light polypeptide	221916_at	1.30E-20	3.57E-17
SCGN	Secretagogin, ef-hand calcium binding protein	205697_at	5.44E-20	1.31E-16
HOXA10	Homeobox a13	213150_at	1.15E-19	2.46E-16
LOXL4	Lysyl oxidase-like 4	227145_at	1.68E-18	3.23E-15
HOXA13	Homeobox a13	231786_at	2.39E-18	3.95E-15
HOXD10	Homeobox d10	229400_at	3.88E-18	5.74E-15
CYTL1	Cytokine-like 1	219837_s_at	7.15E-18	9.83E-15
MYH2	Myosin, heavy polypeptide 2	204631_at	3.76E-16	4.82E-13
HSPB3	Heat schock 27 kDa protein 3	206375_s_at	1.06E-15	1.27E-12
HOXA11	Homeobox a11	213823_at	1.12E-15	1.27E-12
CFC1	Criptic family 1	223753_s_at	4.58E-15	4.90E-12
Genes underexpressed in myxopapillary ependymoma				
APBA2	Amyloid beta (a4) precursor protein-biding, family a, member 2	209871_s_at	6.34E-12	4.36E-09
GPM6A	Glycoprotein m6a	209470_s_at	6.34E-11	3.49E-08
MLC1	Megalencephalic leukoencephalopathy with subcortical cysts 1	213395_at	3.29E-10	1.29E-07
ABTB2	Ankyrin repeat and btb (poz) domain containing 2	213497_at	1.13E-09	3.95E-07
RNF43	Ring finger protein 43	218704_at	1.16E-09	3.99E-07
NCAN	Chondroitin sulfate proteoglycan 3	205143_at	6.37E-09	1.80E-06
KIAA0644	Kiaa0644 gene product	205150_s_at	6.66E-09	1.86E-06
SLC35F1	Solute carrier family 35, member f1	228060_at	1.50E-08	3.96E-06
PKIA	Protein kinase inhibitor alpha	204612_at	1.60E-08	4.11E-06
HNT	Neurotrimin	227566_at	1.99E-08	4.91E-06
CDH4	Cadherin 4	206866_at	2.81E-08	6.68E-06
BIVM	Basic immunoglobulin-like variable motif	222761_at	3.64E-08	8.15E-06
DBI	Diazepam binding inhibitor	202428_x_at	4.86E-08	1.08E-05
GRAMD1B	Gram domain containing 1b	218834_s_at	2.02E-07	3.77E-05
DDR2	Discoidin domain receptor family, member 2	205320_at	2.10E-07	3.89E-05

**Figure 2:**
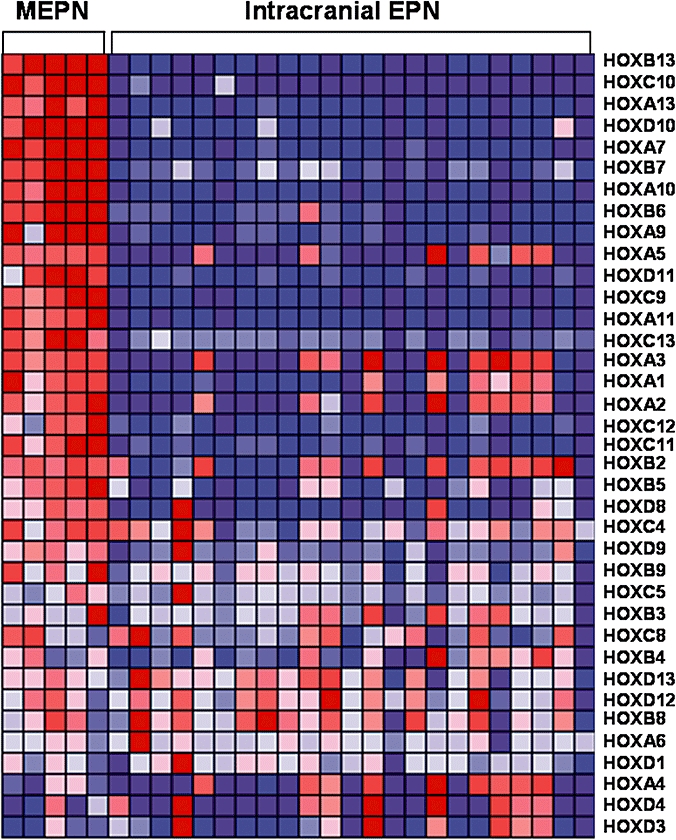
Homeobox family gene expression in pediatric myxopapillary ependymoma compared to pediatric intracranial ependymoma. Data are organized in a heat map format where each row represents a single gene and each column represents an ependymoma sample. Genes are ranked according to difference in expression between MEPN and intracranial EPN. Normalized *z*-scores correlating to the abundance of mRNA relative to a common reference are represented by a color scale where red indicates high gene expression and green indicates low gene expression. Abbreviations: MEPN = myxopapillary ependymoma; EPN = ependymoma; HOX = homeobox.

Three genes of interest were selected for further study. HOXB13 and NEFL were selected because they were among the five most highly expressed genes. PDGFRα was selected due to its potential therapeutic implication. HOXB13 is one of 20 HOX family genes significantly upregulated in MEPN compared to intracranial EPN (891-fold higher, *P* = 7.8E-33). Figure 2 displays homeobox family gene expression in pediatric MEPN compared to pediatric intracranial EPN. Homeobox family genes were chosen for further study due to the large number of highly expressed genes, their well-defined role in embryonic development and emerging literature that they may be involved in oncogenesis. Although multiple HOX family genes were overexpressed, HOXB13 was further examined because it had the highest mRNA expression in MEPN.

NEFL was the fifth most highly expressed gene in MEPN (1098-fold higher, *P* = 1.3E-20). Overexpression of NEFL in MEPN is an unexpected finding, as NEFL is an intermediate filament expressed in neurons. Additional study was conducted to verify and define NEFL protein expression in MEPN. PDGFRα was significantly overexpressed in MEPN (137-fold higher, *P* = 3.8E-5, *q* = 2.1E-3). Although PDGFRα was not among the most highly expressed genes, it was selected and studied due to its potential role as a therapeutic target. PDGFRα is a receptor tyrosine kinase involved in tumor angiogenesis and maintenance of the tumor microenvironment (26). Several PDGFRα inhibitors are U.S. Food and Drug Administration (FDA)-approved.

In addition to MEPN and intracranial EPN, comparative gene expression analysis of HOXB13, NEFL and PDGFRα was expanded to include a variety of pediatric CNS tumors. Of the three genes examined, overexpression of HOXB13 was unique to MEPN, while overexpression of NEFL and PDGFRα was observed in other pediatric CNS tumors. Consistently low HOXB13 mRNA expression was observed in nearly all pediatric CNS tumors including GBM, LCM, MED and PA. ATRT displayed moderate HOXB13 expression; however, expression in ATRT was 315-fold lower than MEPN. NEFL expression varied across different tumor types. Lowest NEFL gene expression was found in intracranial EPN followed by PA and MED. NEFL was moderately expressed in ATRT and GBM and highly expressed in LCM. In addition to MEPN, PDGFRα was highly expressed in PA with little variation. Expression of PDGFRα greatly varied across individual specimens in ATRT, LCM, and MED.

### Immunohistochemical Analysis of HOXB13, NEFL, and PDGFRα Protein in MEPN Compared to Intracranial EPN, SEPN, Spinal EPN, and Adult and Fetal Ependyma

Immunohistochemical staining was performed on 13 MEPN, 13 intracranial EPN, 8 spinal EPN and 5 SEPN. The study numbers were expanded by utilizing adult EPN types as described in the methods. The rarity of SEPN and spinal EPN in the pediatric population also necessitated including adult patients with these types of EPN in the IHC portion of the study. Results of the IHC staining are summarized in Table 2. Immunostaining of HOXB13 was confined to the nucleus. NEFL and PDGFRα staining was cytoplasmic. All three antibodies demonstrated higher protein expression in MEPN than intracranial EPN. No significant differences were detected between adult and pediatric protein expression levels of HOXB13, NEFL, or PDGFRα in MEPN or intracranial EPN. Overall, the upregulation of HOXB13, NEFL, and PDGFRα in MEPN identified by gene expression microarray was recapitulated at the protein level as measured by IHC.

**Table 2:** Summary of immunohistochemical staining of HOXB13, NEFL and PDGFRα. Immunostaining was scored from − to ++ on a subjective scale. Twenty-five percent to 100% of cells staining yielded a score of ++. Very focal staining in individual cells yielded a score of +. Absence of staining was given a − designation. Abbreviations: MEPN = myxopapillary ependymoma; spinal EPN = spinal ependymoma; SEPN = subependymoma; EPN ST = supratentorial ependymoma; EPN IF = infratentorial ependymoma; HOXB13 = homeobox B13; NEFL = neurofilament light; PDGFRα = platelet-derived growth factor receptor alpha.

Diagnosis	HOXB13			NEFL			PDGFRα		
	−	+	++	−	+	++	−	+	++
MEPN	4	1	8	5	1	7	2	0	11
Spinal EPN	7	0	1	6	0	2	2	1	4
SEPN	5	0	0	5	0	0	4	1	0
EPN ST	4	2	0	7	0	0	1	1	5
EPN IF	3	0	0	4	0	1	1	1	4
Adult ependyma	5	0	0	5	0	0	0	4	1
Fetal ependyma	3	0	0	3	0	0	3	0	0

Nine of 13 MEPN were positive for HOXB13, including a single case of incidental adult MEPN found in a filum terminale resected for tethered cord syndrome. All SEPN and infratentorial EPN specimens were negative for HOXB13. Two of six supratentorial EPN were positive for HOXB13 with focal staining of individual cells (+). One of eight spinal EPNs displayed strong staining for HOXB13 (++), while seven of the eight spinal EPN were negative. No staining was identified in the adult spinal cord sections, including the ependyma of the sacral cord, filum terminale or the parenchyma of either of these two sites. The fetal ependyma specimens of 18, 23 and 35 weeks' gestation were negative for HOXB13. Overall, HOXB13 had high sensitivity and specificity for MEPN, especially for cases with the strongest (++) level of staining.

**Figure 3:**
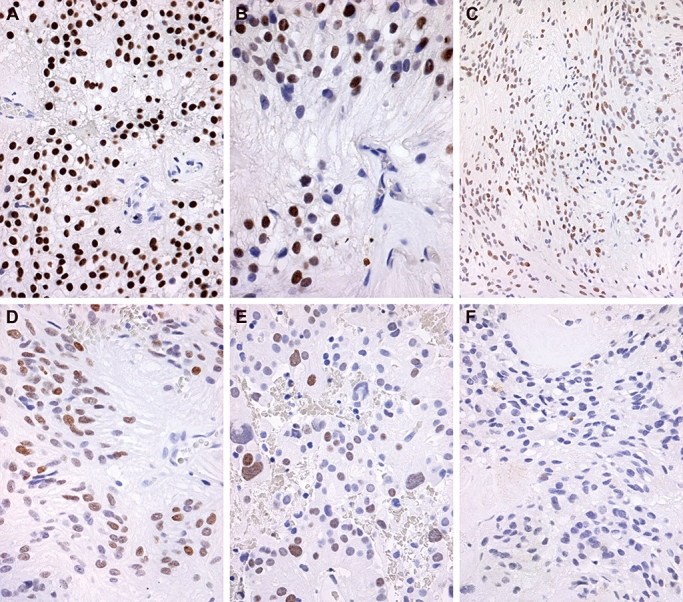
*Representative immunohistochemical staining of homeobox B13 (HOXB13). Immunostaining was scored from − to*++*on a subjective scale. Twenty-five percent to 100% of cells staining yielded a score of*++*. Very focal staining in individual cells yielded a score of*+*. Absence of staining was given a − designation*. **A.** Strong and diffuse nuclear staining of HOXB13, scored as ++, is illustrated in a pediatric myxopapillary ependymoma (MEPN). Immunohistochemistry for HOXB13 with light hematoxylin counterstain, original magnification 400×. **B.** Higher power magnification of the same pediatric MEPN depicted in A demonstrates a perivascular pseudorosette with absence of HOXB13 staining in endothelial cell nuclei, original magnification 600×. **C.** Photomicrograph of an adult MEPN with diffuse expression, scored as ++, albeit with slightly less intensity is shown. Immunohistochemistry for HOXB13 with light hematoxylin counterstain, original magnification 200×. **D.** The same adult MEPN as shown in C is depicted at a higher magnification, original magnification 400×. **E.** Positive HOXB13 immunostaining reaching a score of ++ was identified only in a single adult spinal ependymoma, as illustrated in this photomicrograph. Immunohistochemistry for HOXB13 with light hematoxylin counterstain, original magnification 400×. **F.** Photomicrograph of an adult MEPN with negative HOXB13 staining for direct comparison with A–E. Immunohistochemistry for HOXB13 with light hematoxylin counterstain, original magnification 400×.

Figure 3 displays examples of HOXB13 IHC staining patterns, as detailed in Table 2. Strong and diffuse nuclear staining of HOXB13, scored as ++, in a pediatric MEPN is shown in Figure 3A. In areas with perivascular pseudorosette formation, the ependymoma cytoplasm clearly showed negative immunoreactivity, as did the endothelial cell nuclei of the blood vessel in the center of the pseudorosette (Figure 3B). Strong HOXB13 nuclear immunoreactivity was also found in adult MEPNs, as illustrated by the example with strong and diffuse nuclear staining, scored as ++, illustrated in Figure 3C and D. Spinal EPNs were generally negative for HOXB13, but a single strong (++) case is illustrated in Figure 3E. All negatively stained cases demonstrated crisp and contrasting “positive versus negative” qualities for the commercial HOXB13 antibody used in this study.

Eight of 13 MEPN were positive for NEFL. Absence of NEFL staining was observed in all SEPN and all supratentorial EPN. Two cases of spinal EPN (2/8) and one case of infratentorial EPN (1/5) were scored as strong (++) for NEFL. NEFL was seen only in axons of the spinal cord and filum terminale and not in the ependymal lining of the normal adult specimens. The fetal ependymal lining specimens of 18, 23 and 35 weeks' gestation were also negative for NEFL. In general, IHC for NEFL at the strong (++) level had a high sensitivity for MEPN, with 8/13 cases positive for NEFL compared to 9/13 positive for HOXB13. There was somewhat lower specificity for MEPN with NEFL than with HOXB13, since three non-MEPN EPN variants also showed (++) staining.

**Figure 4:**
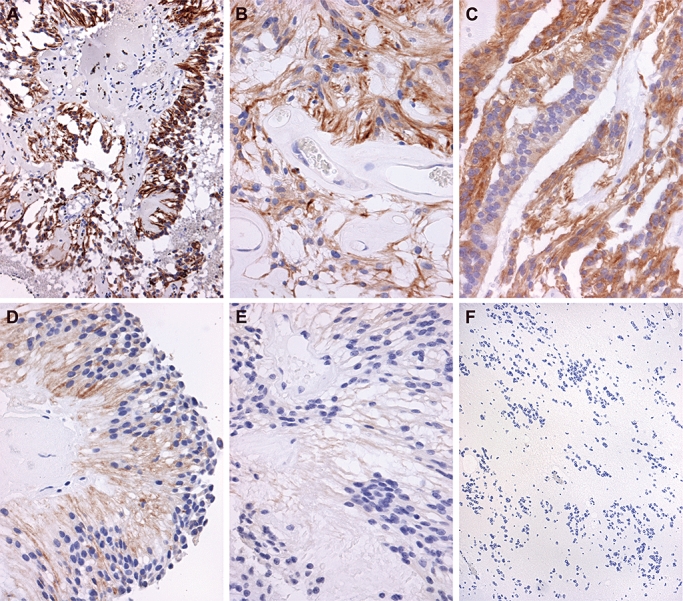
*Representative immunohistochemical staining of neurofilament light polypeptide (NEFL). Immunostaining was scored from − to*++*on a subjective scale. Twenty-five percent to 100% of cells staining yielded a score of*++*. Very focal staining in individual cells yielded a score of*+*. Absence of staining was given a − designation*. **A.** Photomicrograph of a pediatric myxopapillary ependymoma (MEPN) with strong and diffuse cytoplasmic staining in fibrillary areas of tumor, scored as ++. Immunohistochemistry for NEFL with light hematoxylin counterstain, original magnification 200×. **B.** Photomicrograph at higher power shows the perivascular, perpendicularly oriented cell processes surrounding hyalinized blood vessels strongly immunoreactive for NEFL; this adult MEPN achieved a ++ score. Immunohistochemistry for NEFL with light hematoxylin counterstain, original magnification 400×. **C.** Photomicrograph of a pediatric MEPN showing strong, diffuse cytoplasmic staining in areas of the tumor with ependymal, epithelial, non-fibrillary morphological features; this case scored as ++. Immunohistochemistry for NEFL with light hematoxylin counterstain, original magnification 400×. **D.** Photomicrograph of an adult MEPN with ++ diffuse immunoreactivity and slightly less intensity. Immunohistochemistry for NEFL with light hematoxylin counterstain, original magnification 400×. **E.** Focal immunostaining for NEFL in an adult MEPN, yielding a + score. Immunohistochemistry for NEFL with light hematoxylin counterstain, original magnification 400×. **F.** All subependymomas manifested absence of NEFL staining, as illustrated in this adult example. Immunohistochemistry for NEFL with light hematoxylin counterstain, original magnification 200×.

Figure 4 presents examples of NEFL immunostaining patterns. A pediatric and adult MEPN with strong and diffuse cytoplasmic, fibrillary staining are shown in Figure 4A and B, respectively. The pediatric MEPN in Figure 4C shows cytoplasmic staining in areas of the tumor with ependymal epithelial-like features and a non-fibrillary appearance. Figure 4D is an example of an adult MEPN that stained with slightly less intensity, albeit still in a sufficiently diffuse pattern to achieve a score of ++. Figure 4E shows an adult MEPN with focal NEFL staining, yielding a score of +. All SEPN were negative for NEFL, as illustrated by Figure 4F.

All but 2 of the 13 MEPN specimens were positive for PDGFRα. The negative MEPN specimens gave the impression of fixation issues, with extremely weak and edge-related staining that was indeterminate. Four out of the five SEPN were negative for PDGFRα. The ependyma of the filum terminale (specimens taken from five adults without neurological disorders) showed moderate or focal (+) immunoreactivity in 4/5 cases and strong (++) immunoreactivity in the remaining case. Immunostaining was confined to cells in the parenchyma of the adult spinal cord; these cells had a satellite appearance, possibly suggestive of astrocytic cells. No ependymal immunostaining was seen either in adult terminal sacral cord or filum terminale. The fetal ependyma sections were negative for PDGFRα. IHC for PDGFRα demonstrated high sensitivity but poor specificity for MEPN since most infratentorial and supratentorial EPN were positive. Of all the EPN types, the least overlap for PDGFRα was seen with the SEPN group.

**Figure 5:**
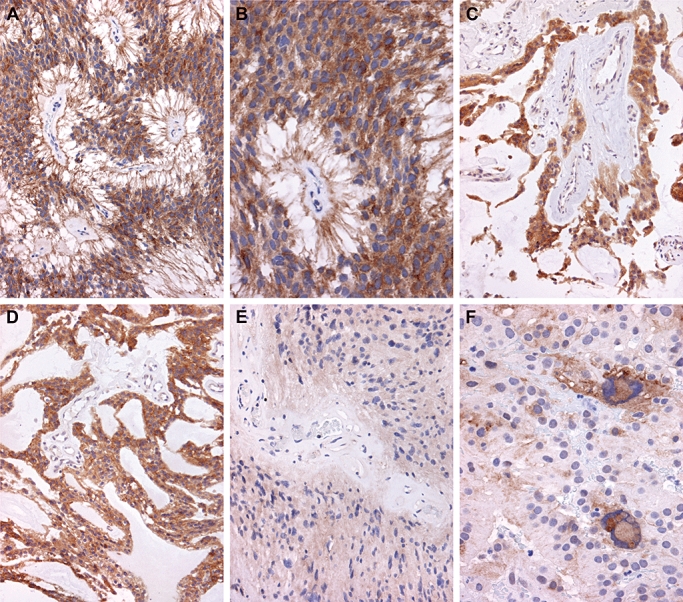
*Representative immunohistochemical staining of platelet-derived growth factor receptor alpha (PDGFRα). Immunostaining was scored from − to*++*on a subjective scale. Twenty-five percent to 100% of cells staining yielded a score of*++*. Very focal staining in individual cells yielded a score of*+*. Absence of staining was given a − designation*. **A.** Low power photomicrograph of a pediatric myxopapillary ependymoma (MEPN) with diffuse immunoreactivity for PDGFRα, scored as ++. Immunohistochemistry for PDGFRα with light hematoxylin counterstain, original magnification 200×. **B.** Photomicrograph at higher power of the same tumor shown in A demonstrates the diffuse cytoplasmic staining throughout all tumor cells of this perivascular pseudorosette. Immunohistochemistry for PDGFRα with light hematoxylin counterstain, original magnification 400×. **C.** Photomicrograph of a different pediatric MEPN also manifests diffuse immunoreactivity, with diffuse staining scored as ++. Immunohistochemistry for PDGFRα with light hematoxylin counterstain, original magnification 200×. **D.** Photomicrograph from a different area of the same case as illustrated in C demonstrates that epithelial-like areas of the MEPN show strong immunoreactivity. Original magnification 200×. **E.** Photomicrograph of an adult spinal ependymoma (EPN) with diffuse, albeit less intense immunoreactivity, scored as ++. Immunohistochemistry for PDGFRα with light hematoxylin counterstain, original magnification 200×. **F.** Photomicrograph of a different example of adult spinal EPN shows strong intensity but a lower staining pattern (++, 400×).

Examples of PDGFRα IHC staining patterns are displayed in Figure 5. Figure 5A and B display a pediatric MEPN with diffuse immunoreactivity, scored as ++. A second pediatric MEPN case with strong, diffuse staining of PDGFRα is shown in Figure 5C and D. Again, immunoreactivity appeared to occur regardless of an ependymal epithelial-like versus fibrillary phenotype, as illustrated in Figure 5C and D. Blood vessel walls and endothelial cells were negative (Figure 5C). The adult spinal EPN in Figure 5E shows diffuse, albeit less intense immunoreactivity, scored as ++. Another case of adult spinal EPN is illustrated in Figure 5F and shows strong intensity, but a lower staining pattern.

## DISCUSSION

Little is known about the biology of MEPN. Clinical trial design requires a better understanding of tumor biology. This is particularly relevant to pediatric MEPN, which have a higher recurrence rate than adult MEPN. The present study uses microarray technology to identify aberrantly expressed genes in pediatric MEPN compared to pediatric intracranial EPN. The overexpression of three genes of interest, HOXB13, NEFL and PDGFRα, was recapitulated by IHC.

Numerous HOX family genes were found to be overexpressed in MEPN compared to intracranial EPN. This finding was verified in part by immunohistochemical staining of HOXB13. As predicted by the microarray data, HOXB13 protein was more highly expressed in MEPN than intracranial EPN. Overexpression of homeobox genes by microarray analysis of mRNA has been previously reported in spinal EPN but not MEPN. A study by Taylor *et al* reported HOX family genes to be overexpressed in spinal EPN from three adults, compared to intracranial pediatric and adult EPN (39). Palm *et al* also reported significant overexpression of HOX family genes in 14 spinal EPN and two EPN from the filum terminale (27). The authors did not stipulate whether their filum terminale examples had MEPN morphology. Although the present study did not include spinal EPN in the microarray analysis, HOXB13 protein expression was not observed in 7 of the 8 spinal EPN. This discrepancy may be due to differences in experimental approach, i.e. protein expression by IHC versus gene expression by mRNA microarray analysis.

The HOX family of genes specifies the patterning of body segments along the anterior–posterior axis by encoding homeodomain transcription factors essential for embryonic development (4, 20). HOX groups 10–13 are associated with the lumbar/sacral region where MEPN arise (43). Accordingly, the four most upregulated HOX genes in MEPN were HOXB13, HOXC10, HOXA13 and HOXD10. Thus, overexpression of HOX groups 10–13 in MEPN may potentially be a function of location. However, normal adult and fetal spinal ependyma and filum terminale were negative for HOXB13 by IHC. Relatively late gestational time periods were available for study (i.e. three fetuses of 18, 23 and 35 weeks' gestation) and HOXB13 might well have been present at earlier gestational time periods. It is probable that HOX groups 10–13 are expressed in lumbar ependyma early in fetal development and then switched off following segmentation. That HOXB13 was not found in normal fetal and adult ependyma suggests that HOX genes are aberrantly expressed in MEPN.

Growing evidence indicates that abnormal HOX gene expression may be involved in oncogenesis. Aberrant HOX gene expression has been noted in acute myeloid and mixed lineage leukemia (9, 13, 18), breast (29), cervical (34), non-small cell lung (28), ovarian (6), prostate (42), skin (24) and thyroid cancers (38). The overexpression of HOX genes in MEPN combined with their potential oncogenic function suggests that the HOX family should be evaluated as a potential therapeutic target. The design of HOX family inhibitors is currently being studied, but is hindered by the functional redundancy of HOX transcription factors and the common co-expression of multiple HOX genes (28).

NEFL was highly overexpressed in MEPN. Neurofilament light polypeptide (68 kDa) is a Class IV intermediate filament expressed in neurons (44). Two homeobox genes that are significantly overexpressed in MEPN compared to intracranial EPN, HOXA3 and HOXA9, have transcription-factor binding sites near the NEFL gene (40). Further research is needed to determine if the concurrent expression of these genes is related. Although the high expression of NEFL is significant to the molecular biology of MEPN, it is a poor therapeutic target due to its ubiquitous expression in axonal processes.

Upregulation of PDGFRα in MEPN suggests that therapeutic targeting of this receptor tyrosine kinase may be an appropriate candidate for future clinical trials. Several PDGFRα inhibitors are FDA-approved, including imatinib mesylate, sorafenib and sunitinib (1–3). Although MEPN response to PDGFRα inhibitors is currently unknown, Fakhrai *et al* reported a case study in which a recurrent spinal ependymoma with positive PDGFRα IHC demonstrated partial remission during treatment with imatinib mesylate (15).

An acknowledged limitation of this study is the relatively small number of pediatric MEPN available for gene expression profiling (n = 5). It should be noted, however, that rapidly frozen surgical specimens are required for this gene expression microarray technique and MEPN are not common in the pediatric age group. Indeed, at the busy tertiary care pediatric hospital where the specimens were obtained, the five MEPN represent virtually every MEPN seen at the institution and accrued over the past 10 years. Thus, it is not surprising that the current study presents the largest cohort to date in the literature that examined pediatric MEPN. The number and diversity of EPN types was increased by including adult patients for the IHC portion of this study, as compared to the gene expression microarray portion of the study which was confined to pediatric MEPN and pediatric EPN. This allowed us to include two adult EPN types that are seldom found in pediatric patients, i.e. SEPN and spinal EPN.

Literature on the molecular biology of MEPN is scarce and impedes progress in the treatment of recurrent tumors. The present study sought to address a specific clinical problem in a grade of tumor that has received relatively little attention despite its ability to recur and disseminate. The study reports the top over- and underexpressed genes in pediatric MEPN that distinguish them from intracranial EPN and identifies at least one potential therapeutic target for recurrent MEPN. Future studies with larger cohorts are needed to confirm our microarray and protein expression findings.

## SUPPORTING INFORMATION

**Table S1.** Cohort demographics including patient diagnosis, age at diagnosis, gender and tumor grade. Grade denotes the histological grade as defined by the World Health Organization classification of tumors of the central nervous system. The table indicates if specimens were included in the gene expression microarray or immunohistochemistry cohorts (Microarray or IHC, respectively). Abbreviations: dx = diagnosis; MEPN = myxopapillary ependymoma; ST EPN = supratentorial ependymoma; IF EPN =  infratentorial ependymoma; SP EPN = spinal ependymoma; SEPN = subependymoma; NE = normal ependyma (spinal cord sections); M = male; F = female; Y = yes; N = no.
