# Supplementary material for: Unique Molecular Characteristics of Pediatric Myxopapillary Ependymoma
Source: Brain Pathol. 2010 May;20(3):560–70. doi: 10.1111/j.1750-3639.2009.00333.x (PMC2871180; doi:10.1111/j.1750-3639.2009.00333.x)
Supplement: Supplementary file 1 [file bpa0020-0560-SD1.rtf]

Dx	Age at Dx (years)	Gender	Grade	Microarray	IHC	
MEPN	12	F	I	Y	Y	
MEPN	13	M	I	Y	Y	
MEPN	17	F	I	Y	Y	
MEPN	13	F	I	Y	Y	
MEPN	12	F	I	Y	Y	
MEPN	35	M	I	N	Y	
MEPN	25	M	I	N	Y	
MEPN	35	F	I	N	Y	
MEPN	69	F	I	N	Y	
MEPN	64	F	I	N	Y	
MEPN	60	F	I	N	Y	
MEPN	62	M	I	N	Y	
MEPN	Adult	NA	I	N	Y	
ST EPN	11	M	III	Y	N	
ST EPN	3	F	II	Y	N	
ST EPN	5	F	III	Y	N	
ST EPN	5	M	III	Y	N	
ST EPN	2	F	III	Y	Y	
ST EPN	18	F	II	Y	Y	
ST EPN	4	F	III	Y	Y	
ST EPN	54	M	III	N	Y	
ST EPN	33	M	II	N	Y	
ST EPN	35	M	III	N	Y	
ST EPN	35	M	III	N	Y	
IF EPN	13	M	II	Y	Y	
IF EPN	6	F	II	Y	N	
IF EPN	13	M	II	Y	N	
IF EPN	7	M	II	Y	N	
IF EPN	2	M	II	Y	Y	
IF EPN	1	F	II	Y	N	
IF EPN	2	M	II	Y	N	
IF EPN	2	M	II	Y	N	
IF EPN	5	M	III	Y	N	
IF EPN	3	M	III	Y	N	
IF EPN	0.5	F	III	Y	N	
IF EPN	6	F	II	Y	Y	
IF EPN	3	M	II	Y	N	
IF EPN	2	M	III	Y	Y	
IF EPN	2	F	III	Y	N	
IF EPN	3	M	III	Y	N	
IF EPN	63	F	II	N	Y	
SP EPN	13	F	II	N	Y	
SP EPN	54	F	I	N	Y	
SP EPN	50	F	I	N	Y	
SP EPN	39	M	II	N	Y	
SP EPN	71	F	II	N	Y	
SP EPN	80	F	II	N	Y	
SP EPN	49	M	II	N	Y	
SP EPN	30	F	II	N	Y	
SEPN	57	M	I	Y	Y	
SEPN	Adult	M	I	N	Y	
SEPN	Adult	F	I	N	Y	
SEPN	Adult	M	I	N	Y	
SEPN	Adult	NA	I	N	Y	
NE	18 week fetus	NA	NA	N	Y	
NE	23 week fetus	NA	NA	N	Y	
NE	35 week fetus	NA	NA	N	Y	
NE	Adult	NA	NA	N	Y	
NE	Adult	NA	NA	N	Y	
NE	Adult	NA	NA	N	Y	
NE	Adult	NA	NA	N	Y	
NE	Adult	NA	NA	N	Y	
